# A prospective analysis of the diagnostic accuracy of 3 T MRI, CT and endoscopic ultrasound for preoperative T staging of potentially resectable esophageal cancer

**DOI:** 10.1186/s40644-020-00343-w

**Published:** 2020-09-10

**Authors:** Jia Guo, Zhaoqi Wang, Jianjun Qin, Hongkai Zhang, Wentao Liu, Yan Zhao, Yanan Lu, Xu Yan, Zhongxian Zhang, Ting Zhang, Shouning Zhang, Nickel Marcel Dominik, Ihab R. Kamel, Hailiang Li, Jinrong Qu

**Affiliations:** 1 Department of Radiology, Affiliated Cancer Hospital of Zhengzhou University, Henan Cancer Hospital, Zhengzhou, 450008 China; 2 Department of Thoracic surgery, Affiliated Cancer Hospital of Zhengzhou University, Henan Cancer Hospital, Zhengzhou, 450008 China; 3 NEA MR Collaboration, Siemens Ltd.,China, Shanghai, 201318 China; 4 Department of Pathology, Affiliated Cancer Hospital of Zhengzhou University, Henan Cancer Hospital, Zhengzhou, 450008 China; 5 MR-Predevelopment, Siemens Healthcare GmbH, 91052 Erlangen, Germany; 6 Department of Radiology, Johns Hopkins University School of Medicine, Baltimore, MD 21205-2196 USA

**Keywords:** Esophageal cancer, Tumor staging, Magnetic resonance imaging, Computer tomography, Endoscopic ultrasound

## Abstract

**Background:**

Patients with esophageal cancer (EC) undergo endoscopic ultrasound and CT based cancer staging. Recent technical developments allow improved MRI quality with diminished motion artifact that may allow MRI to compare favorable to CT for noninvasive staging. Hence the purpose of the study was to assess image quality and diagnostic accuracy of 3 T MRI versus CT and EUS for preoperative T-staging of potentially resectable esophageal cancer.

**Methods:**

Between October-2014 and December-2017, esophageal cancer patients with T-staging by EUS were enrolled in this prospective study. Post-operative histopathologic T-staging was the reference standard. All participants underwent MRI [T2- multi-shot turbo spin echo sequence (msTSE), diffusion-weighted imaging (DWI), and 3D gradient-echo based sequence (3D-GRE)] and CT [non-contrast and multiphase contrast-enhanced CT scanning] 5.6 + 3.6 days after endoscopy. Surgery was performed within 3.6 + 3.5 days after imaging. Two blinded endoscopists (reader 1 and 2) and radiologists (reader 3 and 4) independently evaluated EUS and CT/MRI, respectively. Considering the clinical relevance, patients were dichotomized into early (T1 and T2) vs late (T3 and T4) stage cancer before assessment. For statistical purpose, the binary decision was defined as the ability of the imaging technique to diagnose early stage/not early stage esophageal cancer. Diagnostic performance of EUS, MRI and CT was compared using McNemar’s test with Bonferroni correction; kappa values were assessed for reader performance.

**Results:**

74 study participants (60 ± 8 yrs.; 56 men) with esophageal cancer were evaluated, of whom 85%(63/74) had squamous cell carcinoma, 61%(45/74) were at early stage and 39%(29/74) were at late stage cancer, as determined by histopathology. Intra- and Inter-reader agreement for pre-operative vs post-operative T-staging was excellent for all imaging modalities. Compared to CT, MRI showed significantly higher accuracy for both the readers (reader3: 96% vs 82%, *p* = 0.0038, reader4: 95% vs 80%, *p* = 0.0076, for MRI vs CT, respectively). Further, MRI outperformed EUS with higher specificity (reader 1 vs 3: 59% vs 93%, *p* = 0.0015, reader 2 vs 4: 66% vs 93%, *p* = 0.0081, for EUS vs MRI respectively), and accuracy (reader 1 vs 3: 81% vs 96%, *p* = 0.0022, reader 2 vs 4: 85% vs 95%, *p* = 0.057, for EUS vs MRI, respectively).

**Conclusion:**

For resectable esophageal cancer, MRI had better diagnostic performance for tumor staging compared to CT and EUS.

**Trial registration:**

ChiCTR, ChiCTR-DOD, Registered 2nd October 2014, http://www.chictr.org.cn/showproj.aspx?proj=9620

## Introduction

Esophageal cancer is the seventh most common cancer worldwide, ranking sixth in overall patient mortality [1]. Geographically this global incidence varied by 20 fold, with east Asian males ranking 1st and females ranking 3rd among all other countries [2]. Accurate T staging is necessary for guiding treatment protocols. Surgery is the treatment of choice for Stage T1/T2 (5-year survival rate of 34–36%), while combination of neoadjuvant chemoradiotherapy and surgery is recommended for stage T3/T4a esophageal cancer [3].

Endoscopic ultrasound (EUS) is considered the current standard for preoperative T staging of esophageal cancer [4]. However, it is an invasive procedure that carries a risk of esophageal perforation [5]. In addition, EUS cannot be performed in patients with stenotic lumen and may be limited in the evaluation of advanced tumors due to the finite depth of penetration.

CT remains the most commonly used non-invasive technique in the preoperative T staging of esophageal cancer. Because of poor soft tissue resolution, CT may not differentiate amongst early stage tumors (T1 and T2 lesions) [6]. More recently, multi-detector row CT with dynamic enhanced images has been shown to improve the accuracy of T staging for esophageal cancer [7].

Recent technical improvements in MRI of the chest have resulted in improved image quality. The new technique “multi-shot turbo spin echo sequence” (msTSE), with an acquisition scheme similar to the periodically rotated overlapping parallel lines with enhanced reconstruction, can reduce motion artifacts and bulk motion compared to those of conventional fast spin echo [8, 9]. This is useful especially for uncooperative patients. Diffusion weighted MRI may be useful to characterize the microstructural components of tumors. Previous study on human cadavers has shown that 1.5 T MRI could clearly differentiate the layers of the esophageal wall. Normal muscularis mucosae was seen as a corrugated fine intermediate signal layer surrounded by high-signal-intensity submucosa and the outer low-signal-intensity muscularis propria [10]. 3D gradient-echo based sequence (3D-GRE) is a new 3D gradient-echo sequence, which, due to k-space radial filling and automatic gating technology, can acquire high quality images with improved lesion conspicuity during free breathing [11]. 3D-GRE has a relatively high density of K-space center fill and a relatively low density of peripheral K-space fill, hence the superior contrast and significant improvement of image quality.

We hypothesized that rapid MRI techniques would result in high quality images of patients with esophageal cancer. The purpose of our study was to evaluate the MRI image quality and to compare diagnostic accuracy between MRI, CT and EUS in preoperative T staging of esophageal cancer, differentiating between the early (T1/T2) and late (T3/T4) stage, with histopathologic T staging as the standard of reference.

## Materials and methods

This study was approved by the Hospital Ethics Committee, and all study participants signed the written informed consent for study inclusion.

### Study participants

Between October 2014 and December 2017, consecutive patients with primary esophageal cancer (> 18 years), with preoperative T staging by endoscopy and considered potentially resectable, at the Affiliated Cancer Hospital of Zhengzhou University, were enrolled in this prospective study. A total of 116 study participants met the inclusion criteria and underwent CT (routine clinical procedure to determine the resectability of the tumor) and MRI (study related procedure to determine its diagnostic efficacy) on the same day, 5.6 + 3.6 days after EUS. Based on results of CT scan, the tumor in 42/116 patients were considered non-resectable and were scheduled to receive neoadjuvant chemotherapy, and were thus excluded from the study. The remaining 74 patients constituted our study population and underwent surgery 3.6 + 3.5 days after MRI and CT. Tumor samples that were collected during surgery were fixed in 10% formalin solution and were stained with hematoxylin and eosin following standard tissue processing and staining procedure.

#### EUS procedure

All study participants were required to fast for 6 h before the examination. Post sedation with midazolam and pethidine or fentanyl (Yichang Humanwell Pharmaceutical Co., Hubei, China), EUS was performed by a single endoscopist with 6-year experience in gastroesophageal endoscopy, using a GF-UM2000 (Olympus, Tokyo, Japan) ultrasound machine and ultrasonic probe UM-2R. Study participants were placed in left lateral decubitus position. EUS images were subsequently reviewed and T stage was assigned seperately twice, at a gap of 2 months, by two blinded endoscopists, with 5 and 8 years of experience, respectively. Tumor staging followed the 8th edition of the Union for International Cancer Control-American Joint Committee on Cancer (UICC-AJCC) TNM Classification for esophageal cancer.

### MRI procedure

All study participants were examined on a 3 T MRI scanner (MAGNETOM Skyra, Siemens Healthcare, Erlang, Germany) with an anterior 18-element body coil and in-built posterior 32-element spine coil array. Raceanisodamine hydrochloride (10 mg; Ningbo Dahongying Pharmaceutical Co., Ningbo, China) was injected intramuscularly 15–20 min before MRI in order to reduce peristalsis. All participants were positioned supine with their head-first and were injected 0.1 mmol/kg of gadolinium-DTPA (Consun, Guangzhou, China) through the antecubital vein, at a rate of 2.5 mL/s by MRI-compatible automated injector pump (Spectris Solaris EP, Medrad, Indianola, PA), followed by an equal volume of normal saline solution. The detailed sequence parameters are listed as follows: (1) Scanning the whole chest (sterno-clavicular joint to Lumbar 1 vertebrae) to locate the lesion; post contrast (Gadolinium-DTPA) 3D-GRE: repetition time/echo time = 3.98 ms/1.91 m, thickness = 3 mm, NEX = 1, matrix = 288 × 288, field of view = 300 mm × 300 mm × 72 mm, voxel size = 1.0 mm × 1.0 mm × 3.0 mm, flip angle = 12°, radial views = 1659, scanning time = 309 s. (2) Scanning the lesion to show its detailed wall layers; post-contrast (Gadolinium-DTPA) 3D-GRE: thickness = 1, and rest of the parameters same as above; (3) Scanning the lesion: (A) diaphragm navigation T2-msTSE was performed with respiratory gating: thickness = 3 mm, TR/TE = 5000 ms/97 ms; voxel size = 0.9 mm × 0.9 mm × 3.0 mm, NEX = 1, matrix = 256 × 256, FOV = 240 mm × 240 mm, scanning time = 240 s–360 s; (B) DWI, performed with breath-hold technique: thickness = 3 mm, TR/TE = 5000 ms/55 ms, matrix = 128X128, FOV = 300 mm, scanning time = 157 s, b = 0, 700; (C) post-contrast (Gadolinium-DTPA) 3D-GRE: thickness = 3 mm, TR/TE = 3.98 ms/1.91 m, NEX = 1, matrix = 288 × 288, FOV = 300 mm × 300 mm × 72 mm, voxel size = 1.0 mm × 1.0 mm × 3.0 mm, flip angle = 12 degrees, radial views = 1659, scanning time = 309 s.

### CT scan

All study participants underwent multiphase CT (iCT256 scanner, Philips Healthcare, Amsterdam, Netherlands) scanning immediately after the MRI. All participants were positioned head-first in supine position. After completion of non-contrast scanning 90-100 ml of the contrast agent Ioversol 320 (JIANGSU HENGRUI MEDICINE CO., LTD., Lianyungang, Jiangsu, China) was injected at the rate of 3 ml/s via an anterior elbow vein by an automated injector pump (Spectris Solaris EP, Medrad, Warrendale, PA, USA). The patients were asked to drink a cup of water through a straw immediately before the CT procedure. Covering the view from sternoclavicular joint to Lumbar 1 vertebrae, the arterial phase scanning (optimal for visualization of the tumor) was performed by tracking the aortic peak, followed by the venous (to detect mediastinal lymphadenopathy and distant metastasis) and delayed phase (to determine the benign nature of the stenosis) scanning at 30s and 2 min after the beginning of the arterial phase, respectively. CT scanning parameters were as follows: slice thickness = 5 mm, slice interval = 5 mm, voltage = 120KV, tube current = 300mAs, detector = 128 × 0.625, pitch = 0.993, FOV = 300 mm.

### MR image quality assessment

Assessment of MR image quality was performed by 2 independent radiologists (ZW and JQ with 5 and 20 years of experience in MRI diagnosis, respectively) who were blinded to pathologic T stage, EUS findings and clinical data of the study participants. Each radiologist assigned one of 5 scores for 3 sequence (T2-msTSE, DWI and radial 3D-GRE) images: 5 = excellent, no artifacts, esophageal wall is very clearly visualized; 4 = good, with no artifacts, esophageal wall is clear; 3 = average, with slight artifacts, not affecting evaluation of esophageal wall; 2 = acceptable, with artifacts, may affect evaluation of esophageal wall; 1 = poor, evident artifacts, unable to evaluate esophageal wall. For discrepant scores, the lower score of the two series was considered the final score. If any images scored 1, they were considered poor and hence were to be excluded from the study and while assigning staging on images with score 2, a staging higher than that is visible was considered, according to standard for reporting diagnostic accuracy studies (STARD) checklist [12].

### MRI T staging evaluation

MRI scans were independently evaluated twice, at a gap of 5 months, by two blinded radiologists. T staging was evaluated on T2-msTSE, DWI and 3D-GRE images according to the 8th edition of UICC-AJCC TNM Classification for EC [13], where: T1: tumor invades lamina propria, muscularis mucosae or sub-mucosa T2: tumor invades muscularis propria but without breaking through muscularis propria, T3: tumor invades adventitia, T4a: tumor invades pleura, pericardium, azygos vein, diaphragm or peritoneum; and T4b: tumor invades other adjacent structures, such as the aorta, vertebral body, and trachea.

### CT T staging evaluation

One month after evaluating the MRI scans, the same 2 blinded radiologists independently reviewed the CT scans of the chest, twice at a gap of 5 months, and assigned a T stage for each case. T staging was also performed according to the 8th edition of UICC-AJCC TNM Classification for esophageal cancer [12] as described above.

### Statistical analysis

Sample size was assessed using PASS 11.0, which determined the proportion of discordant pairs as 0.3. From previous imaging studies, the diagnostic sensitivity of MRI and CT were expected to be 88 and 67%, respectively [14–16]. Based on these expected prevalence and sensitivity, two-sided McNemar test, with a significance level of 0.05, provided the need of a sample size of 71 study participants, to yield 90% power (1 minus the probability of a type II error) to detect the significant difference of 0.21 between MRI, CT and EUS.

Statistical analyses were performed using the commercially available SPSS version 22 for Windows (IBM Corp., Armonk, New York, USA). The inter-reader agreement on image quality scores was calculated by kappa test. Further, the inter-reader and intra-reader agreement between pre-operative T staging of esophageal cancer by MRI, EUS and CT with post-operative pathologic findings was evaluated by kappa test, independently. Considering the clinical relevance, the study participants were dichotomized into early (T1 and T2) and late (T3 and T4) stage cancer before assessment. Further, the binary decision (yes/no) was defined as the ability of the imaging technique to diagnose early stage/not early stage esophageal cancer. Compared to the pathological T staging data, sensitivity, specificity and accuracy of EUS, MRI and CT, in T staging esophageal cancer, was determined. The difference between the efficiency of EUS, MRI and CT in T staging of esophageal cancer was analyzed by McNemar’s test. Bonferroni correction was used for adjustment after multiple comparisons, and *P*-values less than 0.0167 (derived from 0.05 divided by 3) were considered statistically significant.

## Results

### Study demographics

**Fig. 1:**
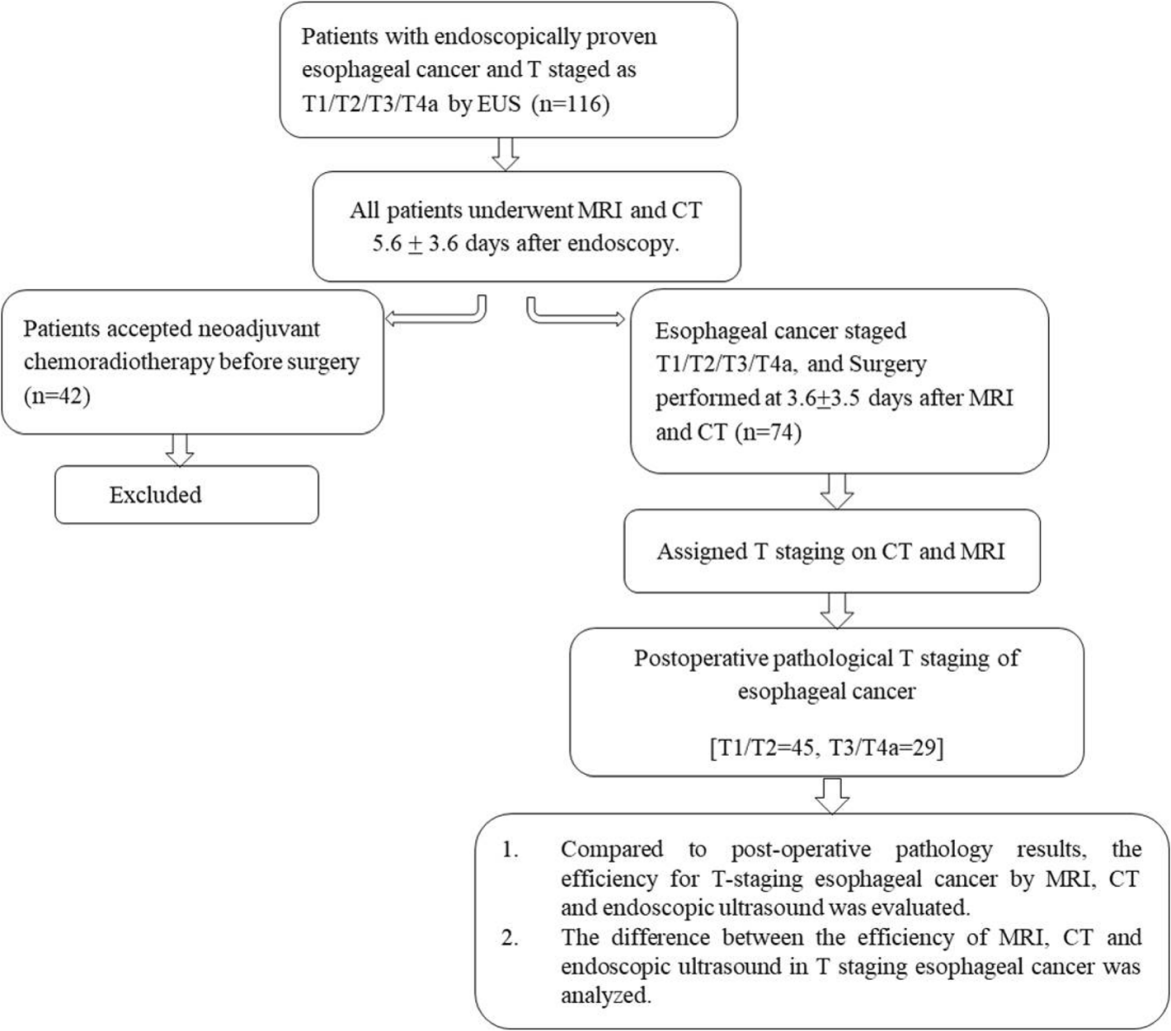
Flow diagram of the study participants and process

The mean age was 60.4 ± 8.2 years; there were 56 men and 18 women. 63 patients had squamous cell carcinoma, 7 had adenocarcinoma and 4 had neuroendocrine carcinoma, as confirmed by post-operative pathology. Pathological T staging confirmed 22 as T1, 23 as T2, 24 as T3, and 5 as T4a (Fig. 1).

### Image quality

As shown in Table 1, the number of MRI scans with score 3, 4 and 5 that do not affect the evaluation of the esophageal wall were 73/74 for reader 3 and 72/74 for reader 4. No cases scored 1 by any reader, while one case for reader 3, and two cases for reader 4, scored 2. Inter-reader agreement of image quality assessment was good (*kappa* = 0.73, *p* < 0.0001).

**Table 1:** MRI quality by MRI readers (*n* = 74)

	Score 1	Score 2	Score 3	Score 4	Score 5
Reader 3	0	1	13	32	28
Reader 4	0	2	12	30	30

### Comparison between EUS, MRI, CT and pathologic staging

Comparison between EUS, MRI and CT with pathologic T staging of early stage (T1/T2) and late stage (T3/T4) esophageal cancer is shown in Tables 2, 3 and 4, respectively. Inter-reader agreement in pre-operative T-staging vs post-operative T-staging was excellent for all imaging modalities: EUS – 60/74 (81%) cases for reader 1 and 63/74 (85%) cases for reader 2 (kappa = 0.80, *p <* 0.0001); MRI – 71/74 (85%) cases for reader 3 and 70/74 (88%) cases for reader 4 (kappa = 0.85, *p <* 0.0001); CT – 61/74 (82%) cases for reader 3 and 59/74 (80%) cases for reader 4 (kappa = 0.90, *p <* 0.0001). Intra-reader agreement was also excellent for all the imaging modalities (kappa = 0.82 to 0.88, *p <* 0.0001).

**Table 2:** Pre-operative EUS T staging and post-operative pathologic T staging in esophageal cancer patients (*n* = 74

Pre-operative EUS T staging		Post-operative pathologic T staging		Sensitivity	Specificity	Accuracy
		T1/T2 (***n*** = 45)	T3/T4 (***n*** = 29)			
**Reader 1**	**T1/T2**	43	2	96% (43/45) (CI: 84.85–99.46%)	59% (17/29) (CI: 38.94–76.48%)	81% (60/74) (CI: 70.30–89.25%)
	**T3/T4**	12	17			
**Reader 2**	**T1/T2**	44	1	98% (44/45)^$^ (CI: 88.23–99.94%)	66% (19/29) (CI: 45.67–82.06%)	85% (63/74) (CI: 74.96–92.34%)
	**T3/T4**	10	19			

^$^ = *p <* 0.016 for EUS vs CT

**Table 3:** Pre-operative MRI T staging and post-operative pathologic T staging in esophageal cancer patients (*n* = 74)

Pre-operative MRI T staging		Post-operative pathologic T staging		Sensitivity	Specificity	Accuracy
		T1/T2 (***n*** = 45)	T3/T4 (***n*** = 29)			
**Reader 3**	**T1/T2**	44	1	98% (44/45) (CI: 88.23–99.94%)	93% (27/29) ^#^ (CI: 77.23–99.15%)	96% (71/74)*^#^ (CI: 88.61–99.16%)
	**T3/T4**	2	27			
**Reader 4**	**T1/T2**	43	2	96% (43/45) (CI: 84.85–99.46%)	93% (27/29)^#^ (CI: 77.23–99.15%)	95% (70/74)* (CI: 86.73–98.51%)
	**T3/T4**	2	27			

* = *p* < 0.016 for MRI vs CT

^#^ = *p* < 0.016 for MRI vs EUS

**Table 4:** Pre-operative CT T staging and post-operative pathologic T staging in esophageal cancer patients (*n* = 74)

Pre-operative CT T staging		Pre-operative pathologic T staging		Sensitivity	Specificity	Accuracy
		T1/T2 (***n*** = 45)	T3/T4 (***n*** = 29)			
**Reader 3**	**T1/T2**	38	7	84% (38/45) (CI: 70.54 93.51%)	79% (23/29) (CI: 60.28–92.01%)	82% (61/74) (CI: 71.83–90.30%)
	**T3/T4**	6	23			
**Reader 4**	**T1/T2**	38	7	84% (38/45) (CI: 70.54–93.51%)	72% (21/29) (CI: 52.76–87.27%)	80% (59/74) (CI: 68.78–88.19%)
	**T3/T4**	8	21			

For statistical purpose, the binary decision (yes/no) was defined as the ability of the imaging technique to diagnose early stage/not early stage esophageal cancer. Hence here sensitivity represents the ability of the imaging technique to diagnose early stage esophageal cancer and specificity represents the ability of the imaging technique to diagnose late stage esophageal cancer. As revealed in Table 2, EUS was good in T staging early stage esophageal cancer, as portrayed by its high sensitivity for both readers; reader 1: 96% (43/45; CI: 84.85–99.46%) and reader 2: 98% (44/45; CI: 88.23–99.94%). However, the low specificity, reader 1: 59% (17/29; CI: 38.94–76.48%) and reader 2: 66% (19/29; CI: 45.67–82.06%), observed in EUS indicated its poor ability in T staging late stage esophageal cancer. The overall accuracy of EUS in T staging esophageal cancer was 81% (60/74; CI 70.30–89.25%) for reader 1 and 85% (63/74; CI: 74.96–92.34%) for reader 2. MRI was better in T staging both early and late stage esophageal cancer, as depicted by its high sensitivity [reader 3: 98% (44/45; CI: 88.23–99.94%) and reader 4: 96% (43/45; CI: 84.85–99.46%)], specificity [reader 3 and 4: 93% (27/29; CI: 77.23–99.15%)] and accuracy [reader 3: 96% (71/74; CI: 88.61–99.16%) and reader 4: 95% (70/74; CI: 86.73–98.51%)]. The data from Table 3 revealed that the CT was decent in T staging both early and late stage esophageal cancer [sensitivity for reader 3 and 4: 84% (38/45; CI: 70.54–93.51%), specificity: reader 3: 79% (23/39; CI: 60.28–92.01%) and reader 4: 72% (21/29; CI: 52.76–87.27%)] and accuracy [reader 3: 82% (61/74; CI: 71.83–90.30%) and reader 4: 80% (59/74; CI: 68.78–88.19%)].

**Fig. 2:**
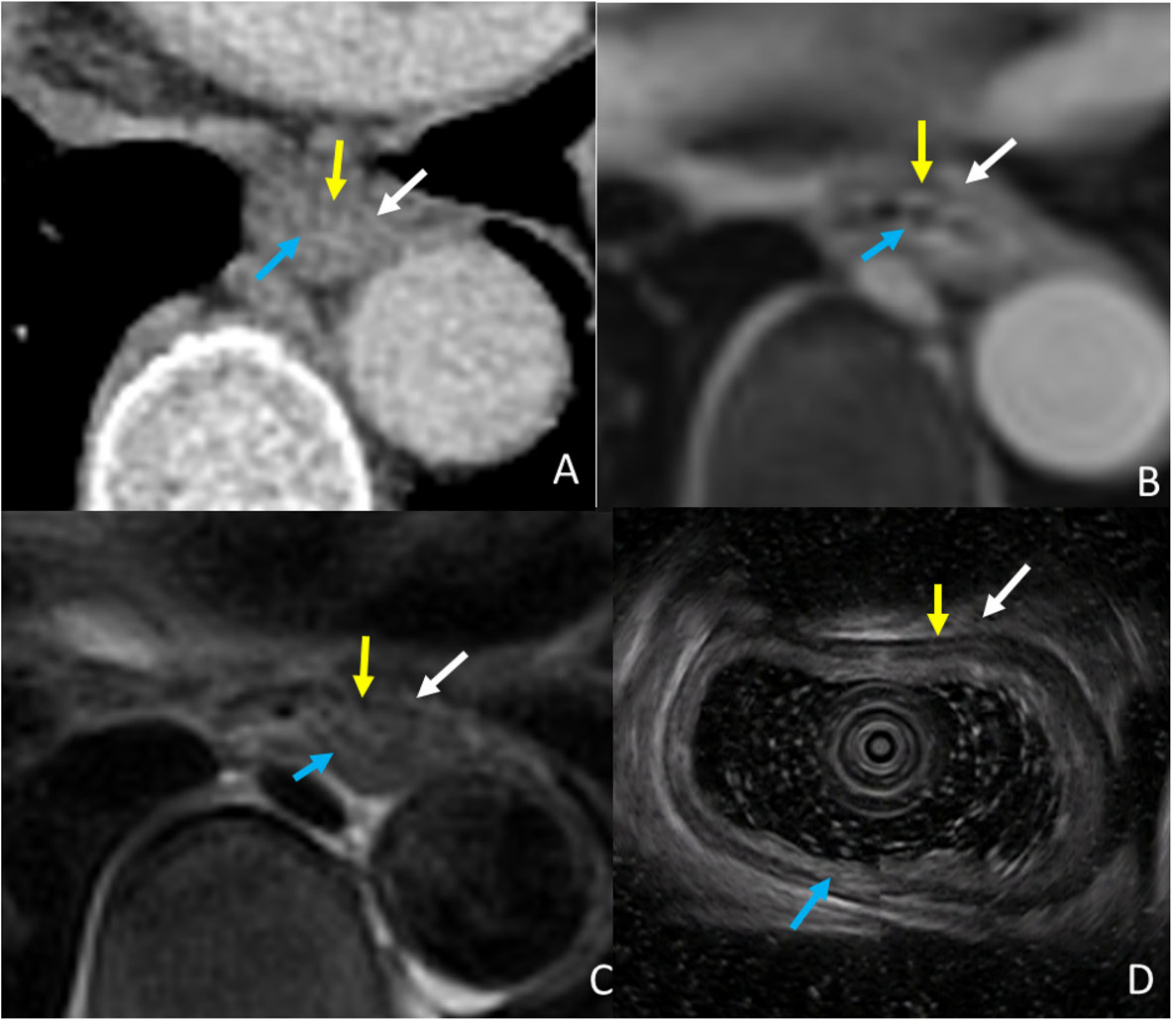
Normal esophagus of a 52-year-old man, demonstrating muscularis mucosae (blue arrow), muscularis propria (yellow arrow) and adventitia (white arrow) in CT (A), 3D-GRE, (B) T2WI-msTSE (C), and EUS (D)

**Fig. 3:**
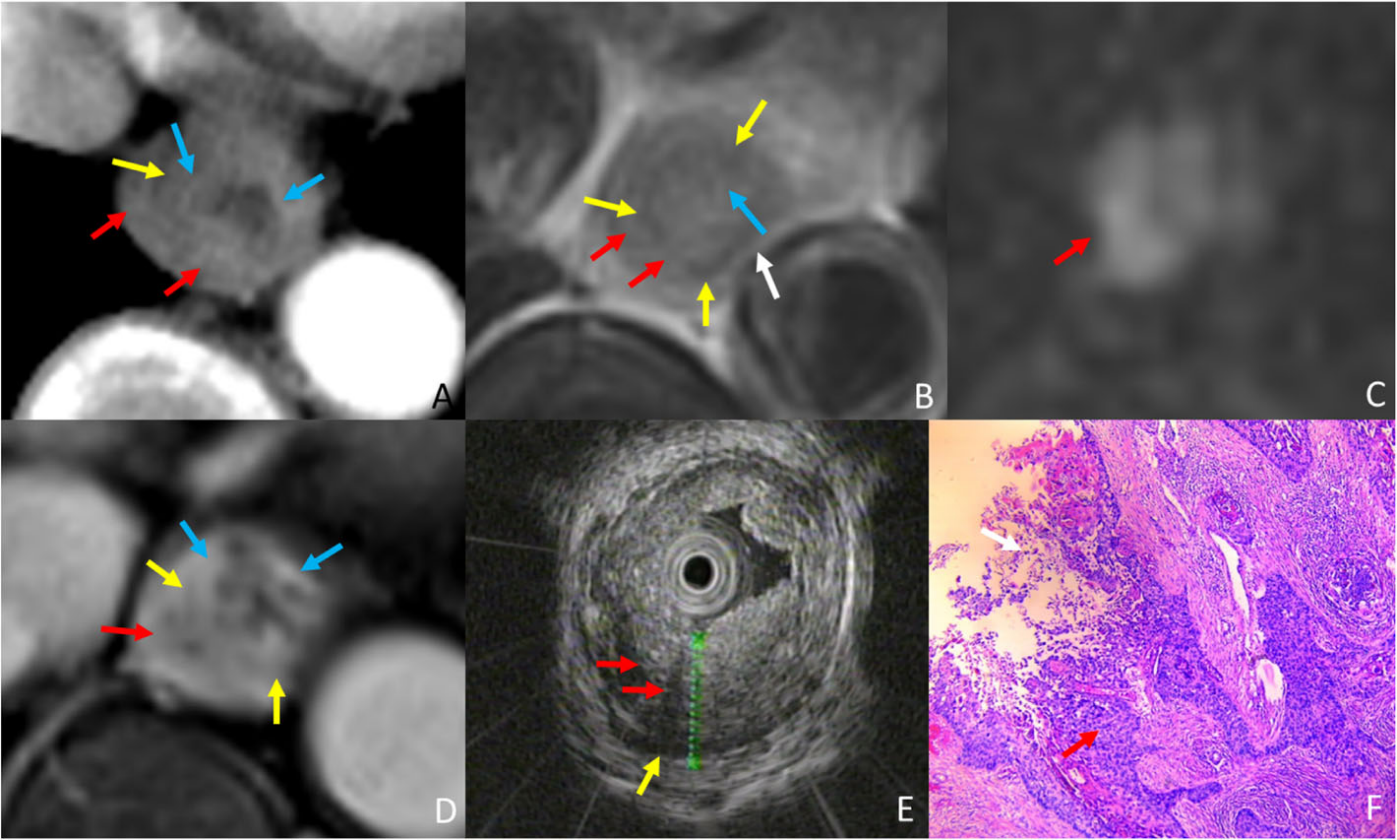
A 74-year-old woman with esophageal squamous cell carcinoma, and late stage (T3) case wrongly diagnosed as early stage (T2) by EUS and correctly diagnosed by MRI.(A) Arterial phase CT shows the high density muscularis mucosae (blue arrow) and muscularis propria (yellow arrow) are interrupted, and the lesion (Red arrow) is staged as T3. (B)T2WI-msTSE shows the lesion (red arrow) invades the adventitia (white arrow). (C) On DWI, b = 700 the lesion (red arrow) is hyperintense. (D) 3D-GRE shows enhanced muscularis mucosae (blue arrow) and the muscularis propria of hypointensity (yellow arrow) is almost disappear, MRI shows lesion (red arrow) in T3 staging. (E) EUS shows tumor (red arrow) invading muscularis propria (yellow arrow) consistent with T2 staging on EUS. (F) H&E stained section at × 40 microscopy confirm that the tumor (red arrow) invades adventitia (white arrow), consistent with T3 stage

**Fig. 4:**
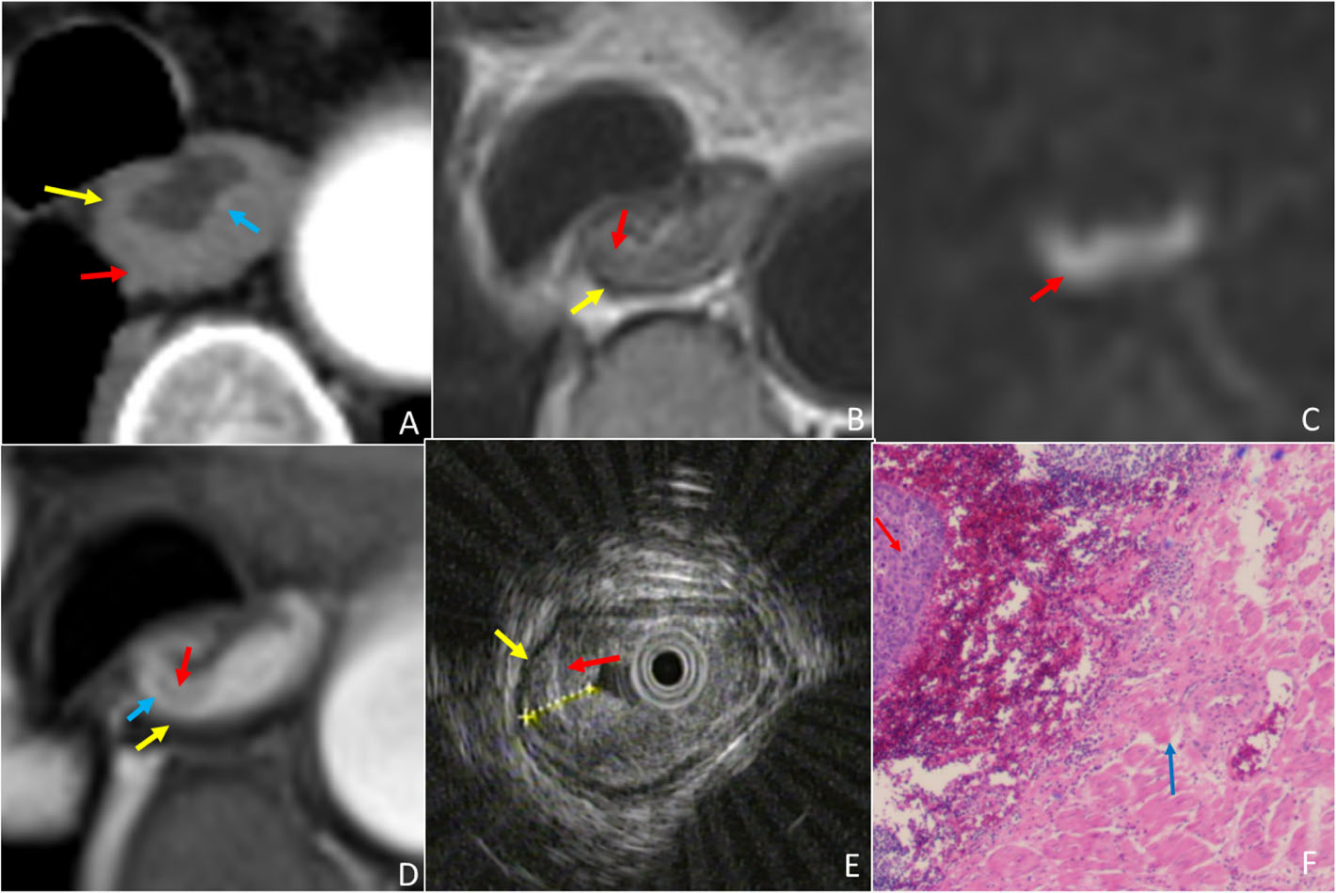
A 67-year-old woman with esophageal squamous cell carcinoma, and Early stage (T1) case wrongly diagnosed as late stage (T3) by CT and correctly diagnosed by MRI. (A) Arterial phase CT shows the partial high density muscularis mucosae (blue arrow) and muscuaris propria (yellow arrow) are interrupted, and the lesion (Red arrow) is staged as T3. (B)T2-msTSE shows lesion (red arrow) with intact hypointensity muscularis propria (yellow arrow). (C) On DWI, b = 700, the lesion (red arrow) is hyperintense. (D) 3D-GRE shows mucosal lesion (red arrow) with enhanced muscularis mucosae (blue arrow) and muscularis propria of hypointensity (yellow arrow), MRI shows lesion in T1 staging. (E) EUS shows tumor (red arrow) invading muscularis mucosae with intact muscularis propria (yellow arrow) consistent with T1 staging on EUS. (F) H&E stained section at × 40 microscopy confirm that the tumor is located in the muscularis mucosae (blue arrow), consistent with T1 stage

**Fig. 5:**
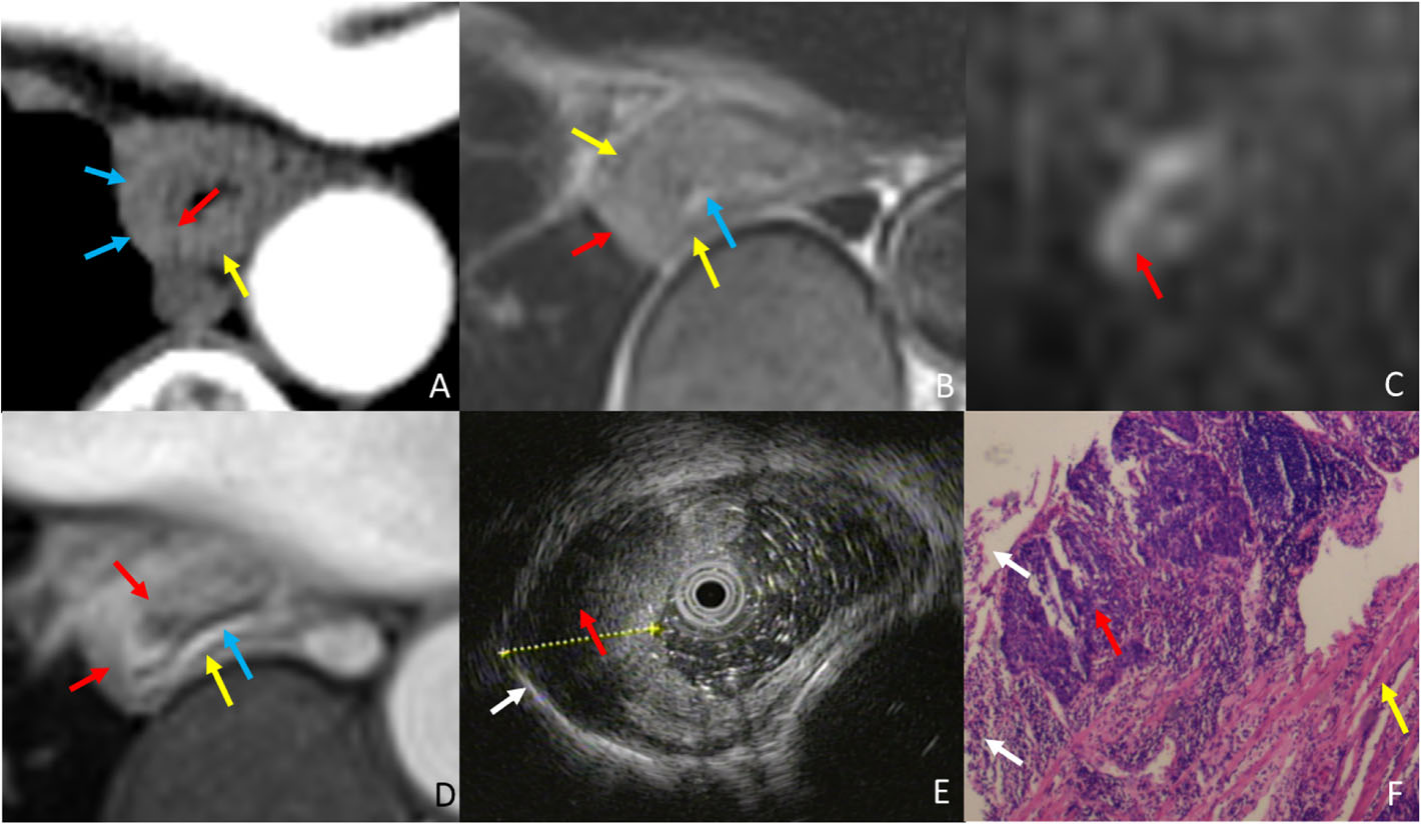
A 51-year-old man with esophageal squamous cell carcinoma, and late stage (T3) case wrongly diagnosed as early stage (T1) by CT and correctly diagnosed by MRI. (A) Arterial phase CT shows the esophageal contour is smooth, the lesion (red arrow) invades the muscularis mucosae (blue arrow) alone, enhanced muscularis mucosae and muscularis propria (yellow arrow) is intact and thus staged as T1. (B)T2WI-msTSE shows the lesion (red arrow) invades the adventitia. (C) On DWI, b = 700 the lesion (red arrow) is hyperintense. (D) 3D-GRE shows enhanced muscularis mucosae (blue arrow) and the muscularis propria of hypointensity (yellow arrow) almost disappear, MRI shows lesion (red arrow) in T3 staging. (E) EUS shows tumor (red arrow) invading adventitia resulting in thinning adventitia (white arrow) and consistent with T3 staging on EUS. (F) H&E stained section at × 40 microscopy confirm that the tumor (red arrow) invades adventitia (white arrow), consistent with T3 stage

**Fig. 6:**
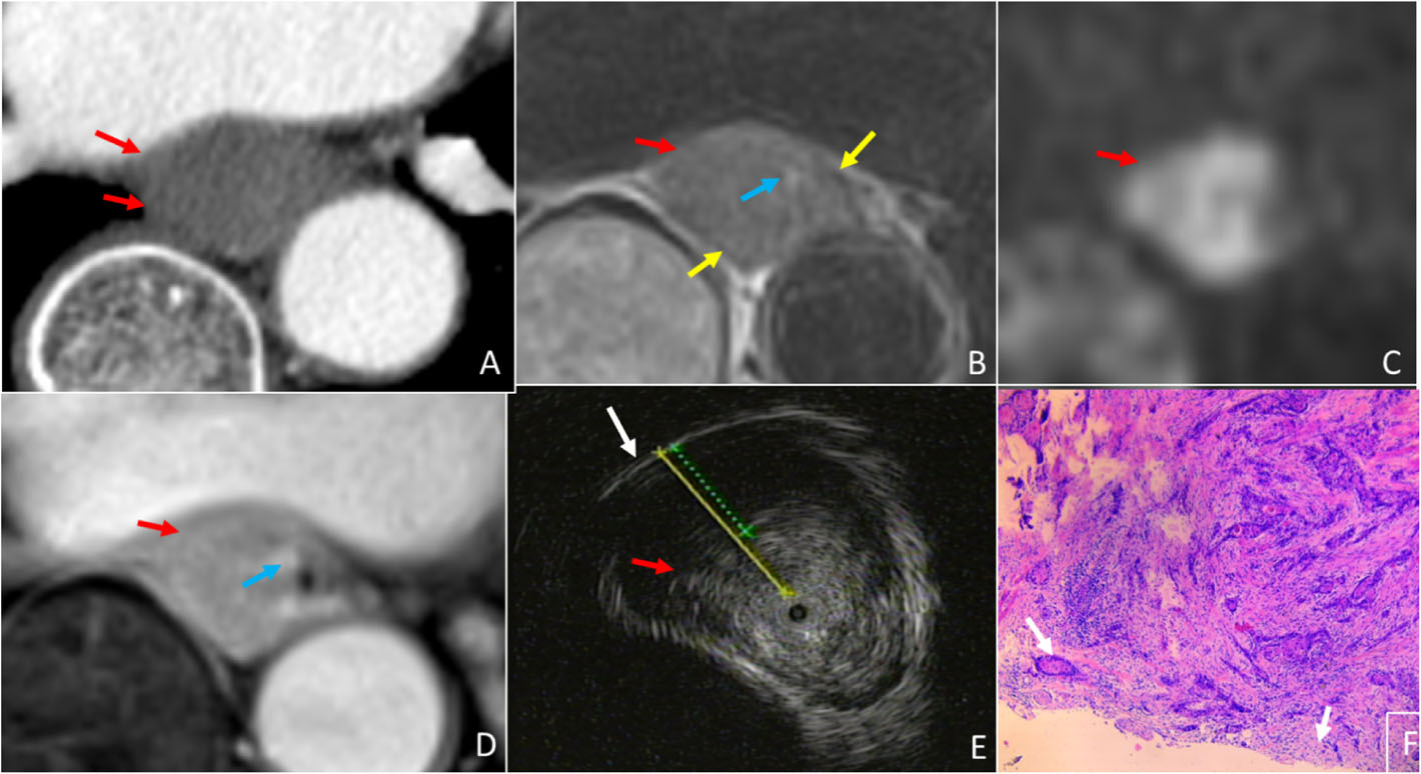
A 65-year-old man with esophageal squamous cell carcinoma, and late stage (T3) case wrongly diagnosed as early stage (T2) by EUS and correctly diagnosed by CT and MRI. (A) Arterial phase CT shows the muscularis mucosae and muscularis propria are interrupted and blurred, and the lesion (red arrow) is staged as T3. (B)T2WI-msTSE shows the lesion (red arrow) invades the adventitia. (C) On DWI, b = 700 the lesion (red arrow) is hyperintense. (D) 3D-GRE shows enhanced muscularis mucosae (blue arrow) and the muscularis propria of hypointensity (yellow arrow) almost disappear, and MRI shows (red arrow) lesion in T3 staging. (E) EUS shows tumor (red arrow) invading muscularis propria with intact adventitia (white arrow) and consistent with T2 staging on EUS. (F) H&E stained section at × 40 microscopy confirm that the tumor (red arrow) invades adventitia (white arrow), consistent with T3 stage

The ability of T staging esophageal cancer amongst EUS, MRI and CT was statistically analyzed. Figure 2 represents the normal portion of the esophagus in all imaging modalities for reference. Though EUS and MRI proved equally sensitive in T staging early stage esophageal cancer, MRI outperformed EUS in T staging late stage cases (Fig. 3), as demonstrated by higher specificity of MRI vs EUS; reader 1 vs reader 3: 59% vs 93%, *p* = 0.0015, reader 2 vs reader 4: 66% vs 93%, *p* = 0.0081, for EUS vs MRI, respectively. Further, MRI also demonstrated better accuracy over EUS for T staging; reader 1 vs reader 3: 81% vs 96%, *p* = 0.0022, reader 2 vs reader 4: 85% vs 95%, *p* = 0.057, for EUS vs MRI, respectively. EUS tends to under-stage late stage cases. Compared to CT, the accuracy of MRI was significantly higher in T staging both early and late stage esophageal cancer (Figs. 4, 5); reader 3: 96% vs 82%, *p* = 0.0038, reader 4: 95% vs 80%, *p* = 0.0076, for MRI vs CT, respectively. EUS was better than CT in T staging early stage esophageal cancer (reader 3 vs 1: 96% vs 84%, *p* = 0.058, reader 4 vs 2: 98% vs 84%, *p* = 0.014, for CT and EUS, respectively). Although CT was relatively better in T staging late stage esophageal cancer (reader 3 vs 1: 79% vs 59%, *p =* 0.057, reader 4 vs 2: 72% vs 66%, *p* = 0.76, for CT and EUS, respectively) (Fig. 6) the difference failed to reach statistical significance. Otherwise, CT and EUS demonstrated a comparable accuracy [reader 1 vs reader 3: 81% vs 82%, *p* = 0.808, reader 2 vs reader 4: 85% vs 80%, *p* = 0.225, for EUS vs CT, respectively] in T staging esophageal cancer cases.

## Discussion

Our study prospectively analyzed 74 study participants with esophageal cancer, 63/74 (85%) of whom were squamous cell carcinoma variant, for the potential of MRI, CT and EUS in T staging of resectable esophageal cancer. MRI was better than EUS in T staging both early and late stage esophageal cancer, and demonstrated higher specificity and accuracy. Further, compared to CT, the accuracy of MRI was significantly higher in T staging both early and late stage esophageal cancer. Despite being the most commonly used imaging modality for staging esophageal cancer, EUS under-staged a very high number of late stage esophageal cancer cases [41% (12/29) and 34.5% (10/29) for reader 1 and 2, respectively]. Compared to EUS, CT was relatively better in T staging late stage esophageal cancer. Otherwise, CT and EUS demonstrated nearly equal accuracy in T staging esophageal cancer cases.

Previous studies have reported squamous cell carcinoma as the most commonly occurring histological type of esophageal cancer, especially in the east Asian countries, unlike the western countries where adenocarcinoma is the most common [2, 14, 15]. EUS is considered the most accurate imaging modality in determining preoperative T staging of esophageal cancer, [15] with reported accuracy rates varying from 64 to 92% [17, 18]. Further, EUS is shown to be insensitive with respect to tumor resectability, the reason for which might be that the criteria based on nodal status and depth of tumor invasion alone is inadequate to preclude surgical resection [19]. CT scan is another explored mode of non-invasive imaging of esophageal cancer that depends on its wall thickening [20], which might not be seen in early stage of the cancer. This explains the 69.2% of the missed-to-demonstrate lesions in a previous study [21], and the reported low T staging accuracy of CT (68.7 to 76.3%) [22].

MRI, when applied to a tubular structure such as the rectum [23], can be highly accurate in evaluating the depth of lesion infiltration. However, motion artifacts have led to the limitation of MRI in the chest. Riddell et al., conducted a study to assess the detection rate of esophageal cancer using high resolution T2-weighted MRI and found that MRI accurately detected only T3 and T4 cases [24]. Other studies showed that the combined T2-weighted and DWI scans detected 33% of T1, 58% of T2, 96% of T3 and 100% of T4 cases of esophageal cancer [25, 26]. Cine MRI is a fast-imaging sequence of true steady-state precession that has been explored to dynamically display esophageal tumor and the risk of its invasion along with its motility [27]. This technology was proved useful to analyze tumor motion and guide in planning the radiotherapy [28].

Our study attempted to measure the accuracy of T staging of esophageal cancer by combining T2-msTSE, DWI and 3D-GRE together, the technical advantages of which, in imaging esophageal cancer, were shown in our previous study [29]. T2-msTSE has an advantage of accurately detecting invasion of adventitia under the fat background and the surrounding vessels with flow void phenomenon. Keeping the degree of background enhancement of lesion lower than that of muscularis mucosae, contrast-enhanced 3D-GRE can give a significantly higher signal of the normal muscularis mucosae, while lowering that of the muscularis propria, which is beneficial in assessing invasion of esophageal cancer into esophageal muscularis mucosae and muscularis propria. Future advances are necessary to improve the technology and adjust the parameters in order to obtain the time-signal intensity curve, a kinetic curve categorizing the perfusion status of gadolinium contrast agent suggestive of benignity or malignancy of the tumor, of the lesion and distinguish lesions of different physiological characteristics.

This study has several limitations. The sequences we tested are not available across different vendor platforms, restricting the repeatability of our work. In this study, EUS was performed before patient recruitment, as inclusion test, while the MRI and CT were performed after the patient recruitment, which can be a source of bias. Here we did not consider T1 versus T2 analysis, in which case EUS may show better performance and clinically useful information. Further, we obtained thicker CT sections (5 mm), which might partly explain the advantage of MRI over CT observed in our study. 3D-GRE requires a longer acquisition time of 309 s, and long-term accumulation of movement (especially the slice direction) may still lead to artifacts. Another limitation is the motion problem in esophageal imaging, thus the 3D-GRE with k-space radial filling and T2-msTSE sequence with diaphragm navigation were adopted here. Furthermore, due to the radial filling property, both 3D-GRE and T2-msTSE sequence were only applied for axial imaging to avoid phase wrap-around. In addition, we did not consider depth of tumor invasion (in mm) as a confounding factor, which may affect the study results, especially related to EUS as it has a poor resolution for deeper lesions.

## Conclusion

Based on these results we propose that, compared to EUS and CT, 3 T MRI (T2-msTSE, DWI and 3D-GRE) shows high diagnostic performance in differentiating early and late stage esophageal cancer, and further studies are needed to confirm this finding.

## Data Availability

The datasets generated during and/or analysed during the current study are not publicly available since they are under the jurisdiction of Affiliated Cancer Hospital of Zhengzhou University but are available from the corresponding author on reasonable request.

## Abbreviations

CT: Computed tomography
*DTPA*: Diethylenetriamine penta-acetic acid
DWI: Diffusion-weighted imaging
EC: Esophageal cancer
EUS: Endoscopic ultrasound
*FOV*: Field-of-view
MRI: Magnetic resonance imaging
msTSE: multi-shot turbo spin echo sequence
NEX: Number of excitations
STARD: Standard for reporting diagnostic accuracy studies
TE: Echo time
TR: Repetition time
TSE: Turbo spin echo
UICC-AJCC: Union for International Cancer Control-American Joint Committee on Cancer
3D-GRE: 3D gradient-echo based sequence
3 T MRI: 3 Tesla magnetic resonance imagin
